# Killing cancer cells by targeted drug-carrying phage nanomedicines

**DOI:** 10.1186/1472-6750-8-37

**Published:** 2008-04-03

**Authors:** Hagit Bar, Iftach Yacoby, Itai Benhar

**Affiliations:** 1 Department of Molecular Microbiology and Biotechnology, The George S. Wise Faculty of Life Sciences, Tel-Aviv University, Ramat Aviv 69978, Israel; 2 Department of Biological Chemistry, Weizmann Institute of Science, Rehovot 76100, Israel

## Abstract

**Background:**

Systemic administration of chemotherapeutic agents, in addition to its anti-tumor benefits, results in indiscriminate drug distribution and severe toxicity. This shortcoming may be overcome by targeted drug-carrying platforms that ferry the drug to the tumor site while limiting exposure to non-target tissues and organs.

**Results:**

We present a new form of targeted anti-cancer therapy in the form of targeted drug-carrying phage nanoparticles. Our approach is based on genetically-modified and chemically manipulated filamentous bacteriophages. The genetic manipulation endows the phages with the ability to display a host-specificity-conferring ligand. The phages are loaded with a large payload of a cytotoxic drug by chemical conjugation. In the presented examples we used anti ErbB2 and anti ERGR antibodies as targeting moieties, the drug hygromycin conjugated to the phages by a covalent amide bond, or the drug doxorubicin conjugated to genetically-engineered cathepsin-B sites on the phage coat. We show that targeting of phage nanomedicines via specific antibodies to receptors on cancer cell membranes results in endocytosis, intracellular degradation, and drug release, resulting in growth inhibition of the target cells *in vitro *with a potentiation factor of >1000 over the corresponding free drugs.

**Conclusion:**

The results of the proof-of concept study presented here reveal important features regarding the potential of filamentous phages to serve as drug-delivery platform, on the affect of drug solubility or hydrophobicity on the target specificity of the platform and on the effect of drug release mechanism on the potency of the platform. These results define targeted drug-carrying filamentous phage nanoparticles as a unique type of antibody-drug conjugates.

## Background

Since the introduction of monoclonal antibodies (mAbs), and the initial clinical trials of antibody therapy in cancer patients, there has been progress in antibody based therapeutics, particularly in oncology. The usage of naked monoclonal antibodies has gradually evolved into drug immunoconjugates. In general drug immunoconjugates are composed of targeting entities (mainly mAbs) chemically conjugated to a cytotoxic drug. The outcome is improved drug efficacy with reduced systemic toxicity. To date, the most clinically-advanced forms of armed antibodies are antibody-isotope and antibody-drug conjugates [1-3]. Key issues in designing and testing immunoconjugates include: 1. the nature of the target molecule, its abundance at the target, whether it is internalizing and at what rate, and its specificity to the target, cells or tissues. 2. the linkers used to attach the drug to the targeting moiety [4]. 3. the drug carrying capacity of the carrier is also a key issue in its potency, thus conjugation schemes, such as the use of branched linkers were devised to maximize the drug payload per target site [5].

A second class of targeted drug delivery platforms are the drug-carrying nanomedicines, such as liposomes, nanoparticles, drug-loaded polymers and dendrimers [6-10]. With a few exception such as targeted liposomes, and antibody-targeted polymeric carriers [11-14], nanomedicines do not utilize a targeting moiety to gain target specificity. Rather, they rely on the "enhanced permeability and retention" (EPR) effect that results from the rapid deployment of blood vessels within rapidly growing tumors resulting in blood vessels in the tumor being irregular in shape, dilated, leaky or defective. As a result, large drug-carrying platforms may gain selective access to the tumor while their exit at non-target sites is limited [10,15,16]. While the immunoconjugates are limited in drug-carrying capacity, usually less than 10 drug molecules per targeting moiety [17], nanomedicines by nature deliver a much larger payload to the target cells. Recently, a novel approach for combining antibody-mediated targeting to cell-surface receptors with a large drug-carrying payload was provided in the description of minicells; enucleated bacteria that are loaded with cytotoxic drugs and targeted using bi-specific antibodies [18].

Filamentous bacteriophages (phages) are the workhorse of antibody engineering and are gaining increasing importance in nanobiotechnology [19-23]. Phage-mediated gene delivery into mammalian cells was developed following studies that identified "internalizing phages" from libraries of phage-displayed antibodies or peptides. [24-31]. Recently, an efficient integrated phage/virus system was developed where tumor targeting and molecular-genetic imaging were merged into an integrated platform [32,33].

Recently we exploited the potential of phages for targeted delivery by applying them as anti bacterial nanomedicines. The phages were genetically engineered to display a target-cell specificity-conferring molecule, up to 5 targeting molecules/phage if displayed on all copies of the phage g3p coat protein. The targeted phages were chemically conjugated, via a cleavable bond to a large payload of an antibiotic, with a maximal loading capacity of more than 10,000 drug molecules/phage [34,35]. The anti-bacterial system was based on drug release at (and not within) the target site. Here we present an evaluation of targeted phage nanomedicines to be applied against cancer cells, with target-mediated internalization followed by intracellular drug release. We show that the growth of target cells can be specifically inhibited when the drug is conjugated either be a covalent bond or through an engineered cathepsin-B cleavage site to the phage coat. Due to the modular nature of the platform, this new class of targeted, drug-carrying viral particles may enable a wide range of applications in biology and medicine.

## Results

### Binding analysis with monoclonal antibodies complexed phage nanoparticles using whole cell ELISA

**Figure 1:**
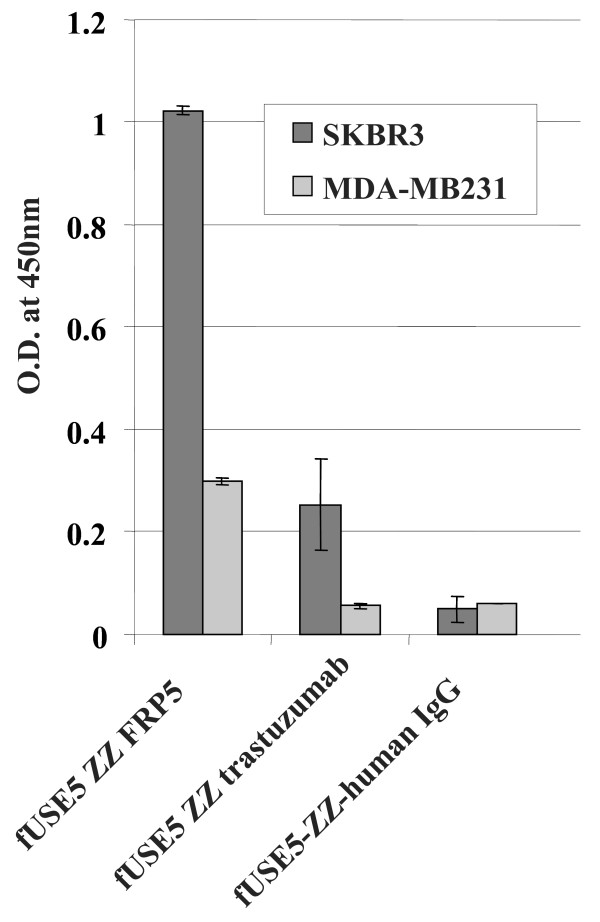
**Comparative binding analysis of antibody displaying phages using whole cell ELISA**. fUSE5-ZZ-chFRP5, fUSE5-ZZ-trastuzumab and control phages fUSE5-ZZ-human IgG were added into wells that contain ErbB2 over-expressing SKBR3 cell line (black bars) or human mammary carcinoma MDA-MB231 cells as control cell line (gray bars) that express a low level of ErbB2. Binding was evaluated by the addition of HRP-conjugated rabbit anti M13 antibodies followed by addition chromogenic HRP substrate TMB. The results were plotted as absorbance at 450 nm. Data represent mean values ± SD of quadruplicate phage samples taking from one of three independent experiments.

The comparative binding analysis was done by whole-cell ELISA as described in Materials and Methods. In order to asses the affect the binding abilities of the different targeting moieties the fUSE5-ZZ, phage vector that polyvalently displays the ZZ domain which enables the phages to form a stable complex with target-specific antibodies was used. The results of this assay are shown in Fig. 1. As shown, the antibody-complexed phages exhibited cell specific binding which corresponded to the level of ErbB2 expression on the target cells, a weak binding to the control MDA-MB231 cell line (that expresses ErbB2 at a low level) was apparent and a much higher binding signal with ErbB2 overexpressing SKBR3 cell line indicating specific binding. No significant signal was obtained when control phages fUSE5-ZZ complexed with normal human IgG were used.

### Evaluation of phage internalization using confocal microscopy

**Figure 2:**
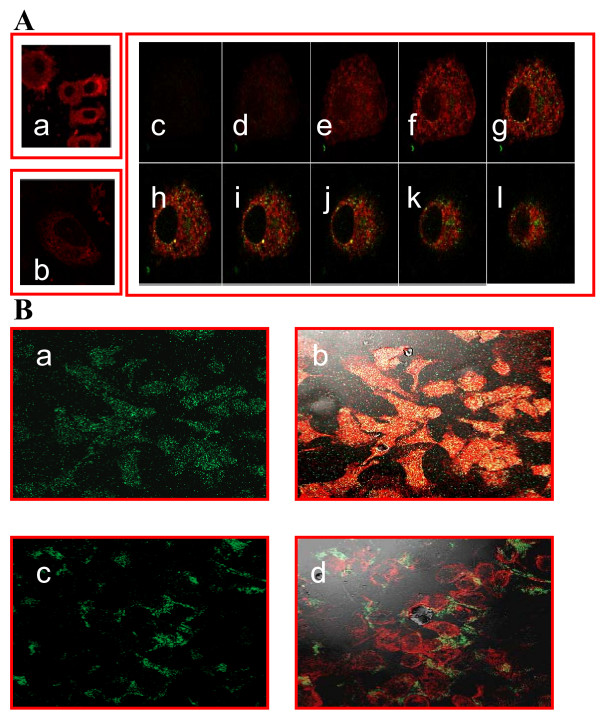
**Evaluation of phage internalization into A431 and SKBR3 cells using confocal microscopy**. **A. Immunofluorescence Staining: (a) **control fUSE5-ZZ-human IgG phages **(b)**, no phage, only antibodies (**c-l) **serial cuts of A431 cells that were treated with fUSE5-ZZ-chFRP5 phages. Membranes were labelled by using red fluorescent CM-Dil cell tracker (MoBiTec, Göttingen, Germany). Phages were detected by monoclonal mouse anti-M13 followed by incubation with FITC conjugated goat anti-mouse IgG (green fluorescence). **B. Evaluation of internalization of FITC labelled, drug conjugated phages into SKBR3 cells using confocal microscopy. a and b **fUSE5-ZZ-chFRP5 phages, **c and d **control fUSE5-ZZ complexed with normal human IgG. As a reference, actin filaments with the cells were labelled by using red fluorescent dye Phalloidin (Sigma, Israel). Phages were labelled directly by FITC. Phages were added to the cells for 2 hr before analysis by confocal microscopy. Panels a and c show the green fluorescence of Fluorescein while in panels b and d the green fluorescence is overlaid on the red fluorescence.

To function as a targeted drug carrying platform, the antibody complexed phages should be efficiently internalized into the target cells. To address this issue, we analyzed the capability of fUSE5-ZZ-chFRP5 complex to internalize into ErbB2 overexpressing human breast adenocarcinoma SKBR3 cells and human epidermoid carcinoma cell line A431 cells using confocal microscopy. A positive internalization signal is typically characterized by bright fluorescence vesicles within the cell cytoplasm together with a decrease in membranous fluorescence. As shown in Fig. 2A, antibody-complexed fUSE5-ZZ-chFRP5 phages where internalized into both types of cells. The orange colour dots that appeared within the cells are possibly the outcome of a combination between the red dyed membrane and the phage marked with green colour generated by the secondary FITC conjugated antibody, which may suggest a lysosomal incorporation of the internalized phages.

### Evaluation of internalization of drug conjugated phage using confocal microscopy

The internalization of antibody complexed phages following drug conjugation was evaluated also following drug conjugation (described below). In this experiment, hygromycin conjugated fUSE5-ZZ complexed with chFRP5 was tested with SKBR3 cells. As opposed to detection with anti phage antibodies as shown in Fig. 2A, here the visualization of hygromycin carrying phages was obtained by conjugating FITC to the phage through a free primary amine found in hygromycin, resulting in green fluorescent labelled fUSE5-ZZ-chFRP5 as previously described [34]. The assay was done in different time points: 2, 12, 24 h, in order to evaluate phage internalization rate. As shown in Fig. 2B, hygromycin carrying phages were internalized into the cells, which could be observed 2 h after adding phages to the cells. In fact, internalization seemed to be maximal when observed at 2 h, and the fluorescent signal diminished during later time point (not shown). In contrast, hygromycin conjugated phages that were complexed with human IgG did not internalize. These results suggested that conjugating a large payload of drug to the phage coat does not seen to inhibit internalization of the antibody-complexed phages, probably occurring through receptor-mediated endocytosis.

### Chemical conjugation of hygromycin and doxorubicin to phage nanoparticles

Conjugation of the two drugs to the phage nanoparticles was done by using EDC chemistry, forming an amide bond between the exposed carboxyl side chains on the phage coat, mostly the ones exposed on g8p, and a free primary amine on the drugs. The drugs we used were hygromycin (an aminoglycoside antibiotics) or doxorubicin (an anthracycline antibiotic). Approximately 10000 molecules of hygromycin were conjugated to each phage by using EDC chemistry as we recently described [34]. EDC reaction causes the formation of a covalent bond between the phage major coat protein, g8p and the drug which does not facilitates a controlled release form of the drug at the target site. However, as shown in Fig. 2, the targeted phage nanoparticles are internalized into the cells possibly entering the lysosomal compartment where they are susceptible to digestion by lysosomal proteases. This led us to the assumption that lysosomal deconstruction of the phage may mediate drug release within the cell.

**Figure 3:**
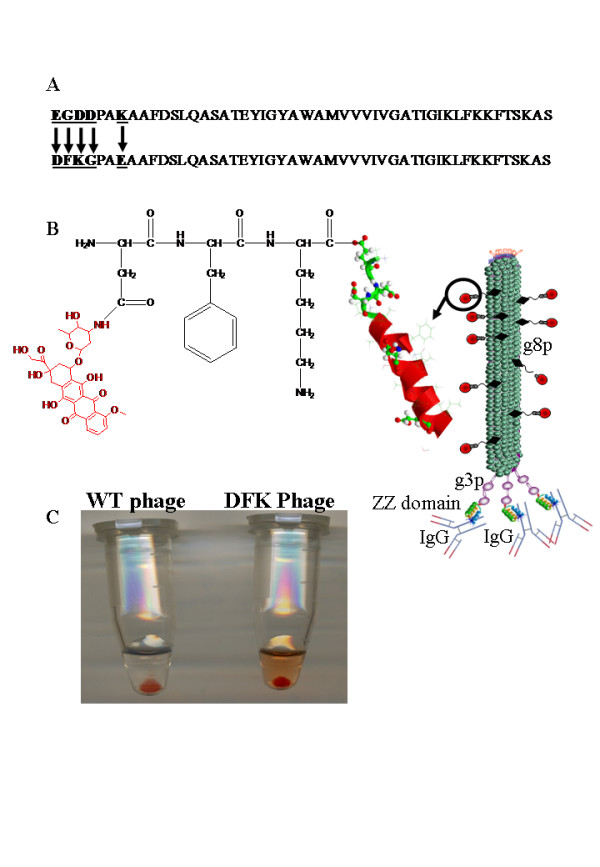
**Controlled release of doxorubicin release from drug-carrying phages**. A. The sequence amino-acid sequence (single-letter code) of the g8p coat protein of fUSE5-ZZ-(p8)DFK phages (top) and native fUSE5 (bottom). The mutated residues are marked by black arrows. B. Drawing (not to scale) of a single fUSE5-ZZ-(p8)DFK phage; In the phage scheme on the right, small turquoise spheres represent major coat protein g8p monomers. Purple spheres and sticks represent the 5 copies of minor coat protein g3p, which is fused to a three-color helix representing the IgG binding ZZ domain. The Y shaped structure represents complexed IgG. An engineered g8p monomer is shown on the left. The helix represents a partial structure of a single major coat protein p8, conjugated through an amino terminal aspartate (D) of the sequence DFK carboxyl side chains a molecule of doxorubicin (red). C. A Photograph of the cathepsin-B release experiment tubes, on the right, doxorubicin carrying fUSE5-ZZ-(p8)DFK phages that was incubated with cathepsin-B, followed by PEG/NaCl precipitation, a reddish soluble D-DOX (verified by HPLC and MS in Fig. 5) is seen as well as a reddish pellet representing the drug conjugated through the internal glutamate residue. On the left is a tube containing fUSE5-ZZ phages that was incubated with cathepsin-B, followed by PEG/NaCl precipitation, the transparent colorless solution indicate no drug release.

To obtain controlled release of the conjugated drug, fUSE5-ZZ-(g8p)DFK phage was designed. fUSE5-ZZ-(g8p)DFK phages display the lysosomal cysteine protease cathepsin-B cleavage site on the phage major coat protein, g8p. In this phage almost all other carboxyl groups on g8p which were susceptible to EDC conjugation were eliminated by site-directed mutagenesis enabling the drug to be released mainly through cathepsin-B activity, a single carboxyl group was left as an internal control for the in-vitro drug release experiments. The sequence of native g8p in comparison with the g8p coat protein of phage fUSE5-ZZ-DFK is shown in Fig. 3A and the scheme of the doxorubicin-loaded phage is shown in Fig. 3B.

### *In vitro *release of doxorubicin from fUSE5-ZZ(g8p)DFK phages

Following doxorubicin conjugation to the phages we evaluated drug release mediated by the lysosomal hydrolase cathepsin-B. As shown in Fig. 3C, drug release could be observed by the red colour of the phage-free supernatant following PEG/NaCl precipitation of cathepsin-B-treated, doxorubicin conjugated fUSE5-ZZ(g8p)DFK phages. In contrast, no drug was released from similarly-treated, doxorubicin conjugated fUSE5-ZZ phages (that do not carry the DFK sequence).

**Figure 4:**
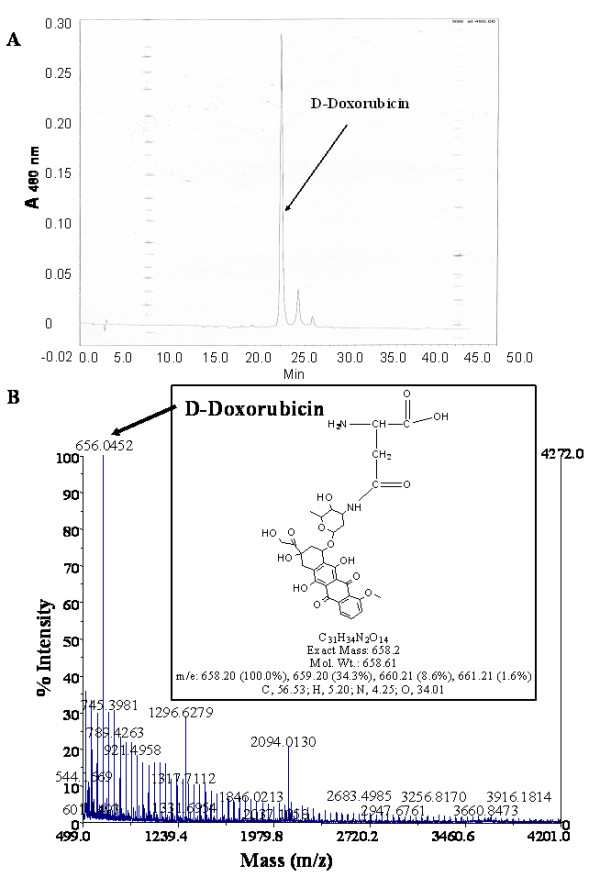
**analysis of the Cathepsin-B released material**. A. The crude cathepsin-B released material from re-suspended, PEG-precipitated, cathepsin-B treated, doxorubicin-carrying fUSE5-ZZ-(p8)DFK phages was analysed using a gradient of acetonitrile in water on a Waters HPLC machine (RP; C-18 column) following the doxorubicin specific emission wavelength 480 nm. Cathepsin-B released material eluted at 24–25 minutes post injection. B. The crude cathepsin-B released material was analyzed by **MALDI TOF MS**. The theoretical mass of the aspartate-doxorubicin (shown in the insert) was observed by the mass spectrometry Analysis as a major peak 656.04 (marked by arrow – corresponding to the weight of aspartate-doxorubicin.

The red supernatant that was recovered from the cathepsin-B-treated, doxorubicin conjugated fUSE5-ZZ(g8p)DFK phages was further analyzed by HPLC at the specific adsorption wavelength of doxorubicin (480 nm). As shown in Fig. 4A, a specific peak corresponding to doxorubicin could be detected. Such a peak could not be observed when fUSE5-ZZ(g8p)DFK phages that do not carry doxorubicin were analyzed under identical conditions. In addition the MALDI-TOF MS analysis of this red supernatant revealed a specific peak corresponding the weight of aspartic acid-doxorubicin adduct, which is the N-terminal amino acid of the displayed DFK peptide Fig. 4B.

### Specific cytotoxicity of targeted hygromycin conjugated phages towards target cells

**Figure 5:**
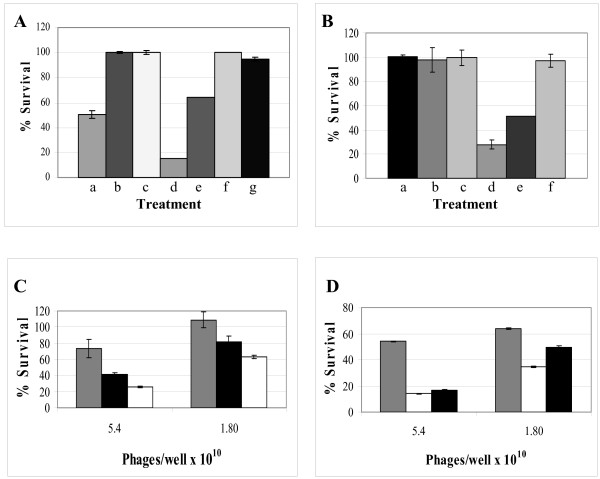
**Toxicity analysis of targeted drug carrying phages by in vitro cell-killing assays**. A) Hygromycin carrying phages on SKBR3 target cells SKBR3 cells were incubated with 5 × 10^11 ^of hygromycin carrying fUSE5-ZZ-chFRP5 phages (a), hygromycin carrying fUSE5-ZZ-human IgG (b), hygromycin carrying fUSE5-ZZ-human IgG (c), 2 mg free hygromycin/well (d), 0.2 mg free hygromycin/well (e), 0.02 mg free hygromycin/well (f) 0.002 mg free hygromycin/well (g). B) Hygromycin carrying phages on HEK293 non-target cells HEK293 cells were incubated with 5 × 10^11 ^of hygromycin carrying fUSE5-ZZ-human IgG phages (a), hygromycin carrying fUSE5-ZZ-trastuzumab phages (b), hygromycin carrying fUSE5-ZZ-cetuximab phages (c), 1 mg/ml free hygromycin (d), 0.1 mg/ml free hygromycin (e), 0.01 mg/ml free hygromycin (f). C) Trastuzumab-targeted, doxorubicin carrying phages on SKBR3 target cells SKBR cells were incubated with serial dilutions of doxorubicin carrying phages; Grey bars, doxorubicin carrying fUSE5-ZZ-trastuzumab, Black bars fUSE5-ZZ-(p8)DFK-human IgG, white bars, doxorubicin carrying fUSE5-ZZ-(p8)DFK-trastuzumab. D) Cetuximab-targeted, doxorubicin carrying phages on A431 target cells A431 cells were incubated with serial dilutions of doxorubicin carrying phages; Grey bars, doxorubicin carrying fUSE5-ZZ-cetuximab, Black bars fUSE5-ZZ-(p8)DFK-human IgG White bars, doxorubicin carrying fUSE5-ZZ-(p8)DFK-cetuximab. The relative number of viable cells was determined using an enzymatic MTT assay and is indicated as the absorption at 570 nm. The results are expressed as percentage of living cells respect to untreated controls. Data represent mean values ± SD of three independent experiments.

Evaluation of the cell cytotoxicity of hygromycin carrying fUSE5-ZZ phages complexed with chFRP5 was done by *in vitro *cell-killing experiments. ErbB2-expressing SKBR3 cells were incubated for 48 h with 5 × 10^11 ^of hygromycin carrying phages, and the relative number of viable cells in comparison with cells grown in the absence of the phage was determined using an enzymatic MTT assay. As shown in Fig. 5A, hygromycin carrying fUSE5-ZZ-chFRP5 phages inhibited target cell growth by 50%, (Fig. 5A treatment a), a >1000 fold improvement in hygromycin potency (in comparison to the free drug). No killing was observed when the cells were treated with, hygromycin conjugated fUSE5-ZZ-phages in complex with human IgG (non-targeted) (Fig. 5A treatment b) or to targeted fUSE5-ZZ-chFRP5 that were not conjugated to the drug (Fig. 5A treatment c). In contrast, the viability of HEK293 cells that were treated with the same dose of hygromycin carrying phages was not affected at all (Fig. 5B).

### Specific cytotoxicity of doxorubicin conjugated fUSE5-ZZ(p8)DFK phages towards ErbB2-expressing cells

Evaluation of the cell cytotoxicity of doxorubicin conjugated fUSE5-ZZ(p8)DFK in complex with the anti ErbB2 antibodies trastuzumab (Herceptin^®^) (Fig. 5C) or anti EGFR cetuximab (Erbitux^®^) (Fig. 5D) was done with *in vitro *cell-killing experiments with A431 and SKBR3 cells. Here the drug was designed to be released in a controlled manner. Doxorubicin was conjugated to the phage using the EDC chemistry, through the engineered DFK tri-peptide where the drugs release is mediated through the cathepsin-B activity in the endosomal-lysosomal compartments. As shown in Fig. 5C, and 5D, doxorubicin carrying fUSE5-ZZ(g8p)DFK in complex with each of the targeting IgGs, but also with the non-targeting human IgG caused efficient killing of the target cells, in a dose-dependent manner. When doxorubicin was conjugated to fUSE5-ZZ phages in complex with trastuzumab (without the DFK sequence), growth inhibition was minimal.

## Discussion

This study presents targeted, drug carrying filamentous bacteriophages (phages) as a drug delivery platform for targeting cancer cells. Our phages represent a modular targeted drug-carrying platform of nanometric dimensions (particle diameter 8 nm, length of a few hundred nm) where targeting moieties, conjugated drugs and drug release mechanisms may be exchanged at will. Specifically, we have generated engineered phages that carried either the drug hygromycin covalently linked to the phage coat, or the drug doxorubicin linked through a cathepsin-B cleavable peptide that was engineered into the major coat protein of the phage. As targeting moieties we used three IgG antibodies; trastuzumab and chFRP5 that target ErbB2 and cetuximab that target the EGFR. When target cells were treated with the targeted drug-carrying phages, selective cell killing could be demonstrated with a potentiation factor of up to several thousand over the free drug.

In our study we used EGFR and ErbB2 as targets; both are very well characterized in the field of targeted anti cancer therapy. In fact, two of the three antibodies we used as targeting moieties are used clinically to treat cancer patients (trastuzumab and cetuximab). All three antibodies we used were shown before to facilitate the delivery of cytotoxic payloads to target cells and tumor models [36-41]. In addition, antibody-displaying filamentous phages have been shown to undergo internalization into target cells, which laid the foundation for proposing to use such "internalizing phages" as gene delivery vehicles [26,29,32,33,42,43].

The drug carrying capacity of the platform is a key issue in its potency. With antibody-drug conjugates, the amount of cytotoxic drug that can be conjugated to the antibody is usually limited by the conjugation chemistry that, if pushed to the upper limit, may reduce its capacity to bind antigen. As a result, such conjugates carry no more than 8 drug molecules per mAb [17]. Recently more elaborate drug conjugation schemes, such as the use of dendrimers and branched linkers to increase carrying capacity were devised to maximize the drug payload per targeting molecule that binds a target site [44-46]. Our phages carry as much as 10^4 ^drug molecules/phage which maximizes the intracellular drug load upon internalization of the platform into the target cells.

Considering the linkers used to attach the drug to the targeting moiety, an ideal linker should be stable in the serum and readily degraded within the intracellular milieu. Some examples from the field of antibody-drug conjugates are acid-labile linkers and enzyme-cleavable linkers [4]. We chose to evaluate two approaches; direct covalent conjugation of the drug to the carrier and conjugation of the drug through an engineered cathepsin-B cleavage site. Our results (Fig. 5) show that both approaches are viable, but the results may vary with different drugs and/or targeting antibodies. Phages that were used to deliver covalently linked hygromycin to ErbB2 expressing cells (chFRP5 IgG as the targeting moiety, Fig. 5A) could cause specific cell growth inhibition. When phages were used to deliver doxorubicin to either erbB2 expressing cells (trastuzumab as the targeting moiety, Fig. 5C) or EGFR expressing cells (cetuximab as the targeting moiety, Fig. 5D), phages to which the drug was linked covalently were inefficient in inhibiting cell growth, while phages that carried the cathepsin-B releasable drug were more efficient, suggesting that with this particular phage-drug combination, an engineered drug release mechanism is necessary to maximized potency. We could not link hygromycin to DFK-displaying phages, since we found that hygromycin with a single amino-acid adduct (as is the product of cathepsin-B release drug in our system) is inactive as a drug (data not shown).

Early antibody-drug conjugates were comprised of a monoclonal antibody covalently linked to several molecules of a clinically used anti-cancer drug. The linker connecting the antibody and the drug was either non-cleavable or cleavable upon entry into the cell. In the early development phase of antibody-drug conjugates, it was believed that the tumor specificity of anti-cancer drugs could be improved merely by linking these drugs directly to antibodies via amide bonds [17]. In most cases, the conjugates lacked cytotoxic potency and were less potent than the un-conjugated drugs [47]. Only in the past few years the critical parameters for optimization have been identified and have begun to be addressed. These include low drug potency, inefficient drug release from the mAb and difficulties in releasing drugs in their active state [44]. On the basis of this much research has been focused on designing new linker technology. The use of peptides which are susceptible to enzymatic cleavage, as conditionally stable linkers for drugs to mAbs. The peptides are designed for high serum stability and rapid enzymatic hydrolysis, once the mAb-drug conjugate is internalized into lysosomes of target cells. For example cathepsin-B sensitive peptides. Cathepsin-B is a cysteine protease found in all mammalian cell lysosomes. The cathepsin-B cleavable di-peptide Phe-Lys was used for conjugating doxorubicin to BR96 mAb which were previously conjugated through a hydrazone labile linker [48]. The resulting immunoconjugate showed levels of immunological specificity that had been unobtainable using the corresponding hydrazone-based conjugates.

The objective of the experiments described herein was a feasibility study of applying targeted drug delivery as an anti cancer tool. The system was designed on three key components: 1) a targeting moiety, exemplified here by various monoclonal tumor specific antibodies complexed via the ZZ domain [34]. 2) A high-capacity drug carrier, exemplified here by the filamentous phage, with its 3000 copies of major coat protein, each amendable to drug conjugation. 3) A drug linked directly or through a labile linker that is subject to controlled release, exemplified here by hygromycin conjugate directly or by doxorubicin that was linked through a cleavable peptide expressed on all copies of phage major coat protein. In the case of covalently-linked hygromycin, we postulate that a partial non selective release in the lysosomes post internalization would eventually lead to a specific killing of target cell. Several features led us to use hygromycin as the model drug: The first was the simplicity in which hygromycin can be conjugated to the phages through simple EDC chemistry. Hygromycin has two primary amino groups, one for phage conjugations and the other for drug or analyte conjugation (such as FITC as we have report previously) [34]. Another important feature at this stage is the high drug solubility in water. With this chemistry, a carrying capacity in excess of 10^4 ^drug molecules/phage was previously reported by us [34].

The second example was a controllable release mechanism that was genetically engineered into the phage major coat protein g8p (p8). We mutated the N-terminus of p8 to express a cathepsin-B cleavage peptide with the sequence of DFK [48]. Aspartate (D) was added to the sequence FK for the creation of two options for chemical conjugation; through the α-amine or through the carboxyl side chain. In addition to this mutation, the native aspartic side chains were mutated to non carboxyl side chains (asp 11 (Fig. 3A) was retained since it is buried within the phage coat an inaccessible to conjugation [49]). The native lys8 was mutated to glu7 in the newly mutated p8 and used as internal control for drug conjugation and to maintaining balanced number of charged residues that are important for phage solubilisation. Indeed, from Fig. 3C one may appreciate that there was partial release of doxorubicin upon cathepsin-B treatment, since doxorubicin molecules linked to glu7 were not released. Since two of the native carboxyl residues asp4 and asp5 were deleted it is important to note that by this genetic modification in the structure of the major coat protein-8 we have reduced the potential drug capacity by more then 60%.

Doxorubicin was used as a model drug; primary by two reasons; the first is its reporter properties, fluorescence as well as specific emission in the wavelength of 480 nm. This property was helpful for monitoring of drug release. The second is the relative tolerance for conjugation of linkers into the single primary amine located to the aminoglycoside ring tailored specifically for the solubilization of this highly hydrophobic drug. Doxorubicin was conjugated to phages through EDC chemistry, resulting with reddish solution. The releasing experiment with commercial cathepsin-B led to a complete release of all connected doxorubicin molecules. Each DFK phage release about ~3500 doxorubicin molecules as we measured by a specific reading at 480 nm with a reference of a calibration curve of free doxorubicin. This results correlates with the maximal theoretical capacity of this phages. Using HPLC analysis, we found that the release was limited to the DFK phage only, while doxorubicin linked covalently to the wild-type phage coat was not released. Further MALDI-TOF MS analysis showed that the released moiety was doxorubicin with aspartate linked to it. Such adducts are common when labile linkers are used do deliver drugs, and in the case of doxorubicin, do not seem to inactivate it. This is similar to the released drug of the "non-cleavable" antibody-drug conjugates where upon degradation in the lysosomal compartment, the drug remains covalently linked to a single amino acid, either lysine for maytansinoid conjugates or cysteine for auristatin conjugates [50-52].

Internalization of the phages, unconjugated or armed with drug could be demonstrated by fluorescence confocal microscopy (Fig. 2). The result of the target cell killing assays showed that the soluble drug hygromycin connected through a non cleavable stable amide bond, directly to phage coat carboxyl residues, could achieve impressive potency improvement, in factor of >1000 over the free drug (Fig 5A) (5 × 10^10 ^phages carrying 10^4 ^drug molecules/phage, carry 0.43 μg free drug, which inhibits cell growth as well as ~1 mg of free drug). This occurred although poor drug release within the cells could be expected.

The interpretation of the results of doxorubicin-carrying phages is more complex, since the goal of this system was a proof of new concept for the construction of cleavable linkers by genetic engineering instead of conventional organic chemical linkage. Our results show the DFK peptide to be specifically cleaved by cathepsin-B, specifically at the engineered site (Fig. 3) and within the target cells. Further, doxorubicin-carrying DFK phages (Fig. 3B and 3C, white bars) are more potent in comparison the phages that carry covalently-linked doxorubicin (Fig. 3B and 3C, grey bars) even though the latter carry 10000 drug molecules/phage while the former carry 3500 drug molecules. Moreover, phages to which doxorubicin was covalently linked, although they were target-specific, inhibited cell growth less efficiently than non-specific, human IgG linked DFK phages, further demonstrating the contribution of the engineered drug-release mechanism to the potency of the platform. As for the limited specificity of doxorubicin-carrying DFK phages, we have already observed that coating phages with a high density of hydrophobic molecules limits the solubility of the phages [34] and cases them to become "sticky", that is, to bind non-specifically to bacteria and to cells (unpublished results). The results shown in Fig. 5B and 5C suggest that this may also be the case with doxorubicin-carrying phages, since comparably levels of cell-killing could be observed when that phages were linked to the targeting antibodies, or to the irrelevant control, normal human IgG. Such non-specific killing could not be observed with hygromycin-armed phages (Fig 5A). Doxorubicin is known as a drug of which doses are limited by unwanted toxicity to non-tumor tissues [53]. Doxorubicin and other anthracyclines are amphiphilic molecules known to interact with cell membranes [54], which may cause non-specific binding. Large polymer-doxorubicin conjugates were reported as having limited solubility [55], which may also affect target specificity. One may concluded that drug delivery platforms that carry the drug on the outside will be limited to highly water soluble drugs that do not bind non-discriminately to cells. However, a remedy to this limitation may be found in linking the hydrophobic drug to the phage through a solubility-enhancing linker, as we have recently reported [34]. We have shown that the potency and the target specificity of anti bacterial chloramphenicol-armed phages was substantially improved when this hydrophobic drug was linked to the phage coat through small hydrophilic molecules that served as solubility-enhancing linkers. On the other hand, phages that were directly armed with the hydrophobic drug chloramphenicol were less specific [34]. An additional advantage of such an added hydrophilic coat is that it reduces both the immunogenicity and antigenicity of the drug-carrying phages upon injection into mice (unpublished data).

## Conclusion

To conclude, our study demonstrated a proof of principle of targeted, drug-carrying filamentous bacteriophage as anti cancer agents. The issues of Pharmacokinetics, biodistribution and immunogenicity and tumor penetration: these parameters are key issues in current phage therapy studies [56,57]. Basically, phages are immunogenic on one hand, and upon intravenous injection are removed quickly by the reticuloendothelial system on the other. Attempts to modulate phage pharmacokinetics were based on isolating long-circulating mutants of phage lambda [56]. But no such studies were done with the filamentous phages. We believe that chemical modification of the phage coat (as we do during drug conjugation) should modulate the pharmacokinetics, the biodistribution and the immunogenicity in comparison to bare phages. Regarding tumor penetration, phages can not be regarded as huge complexes as may be evident from their molecular weight which is about 15 million dalton. Due to their needle-like structure, they may very well penetrate into tumors, as may be suggested by the study where xenografts in nude mice were eradicated following IV injection of phages that delivered a therapeutic gene [32]. We are currently comparing the immunogenicity, pharmacokinetics and biodistribution of un-conjugated to drug-carrying phages in animal studies.

## Methods

### Cell lines

Cell lines used were the human breast carcinoma cell lines SKBR3 and MDA-MB231, the human epidermoid carcinoma A431 cell line, and the human kidney HEK293 cell line. Cells were maintained in Dulbecco's modified Eagle medium (DMEM) containing 10% foetal calf serum (FCS), unless mentioned otherwise.

All the chemicals used were of analytical grade and were purchased from Sigma (Israel). Unless stated otherwise, reactions were carried out at room temperature (about 22°C).

### Linking phages to the targeting antibodies

Phage fUSE5-ZZ that can be complexed with IgGs was recently described [35]. Briefly, this is a derivative of the filamentous phage vector fUSE5 [58] that was engineered to display the IgG Fc-binding ZZ domain on all copies of the g3p minor coat protein. Filamentous phages were routinely propagated in DH5-αF' cells using standard phage techniques as described [59]. Phages were usually recovered from overnight 1 litre cultures of carrying bacteria. The bacteria were removed by centrifugation and the phage-containing supernatant was filtered through a 0.22 μm filter. The phages were precipitated by addition of 20% (w/v) polyethylene glycol 8000 PEG/2.5 M NaCl followed by centrifugation as described [59]. The phage pellet was suspended in sterile miliQ double-distilled water (DDW) at a concentration of 10^13 ^pfu/ml and stored at 4°C.

To form a complex with targeting antibodies, 10^12 ^phage in 1 ml PBS were mixed with 1.6 μg of the IgG: chFRP5 [36], Herceptin^® ^(trastuzumab, Genentech, USA), Erbitux^® ^(cetuximab, ImClone, USA) or control normal human IgG (Sigma, Israel). The phage-IgG mixtures were left for at least 1 h at room temperature. This phage to IgG ration yields occupancy of about 50% of the available ZZ sites on the phage [35].

### Construction of phage fUSE5-ZZ-(g8p)DFK

fUSE5-ZZ was further modified to display the lysosomal cysteine protease cathepsin-B cleavable DFK tri-peptide [48] on all copies of the g8p major coat protein. The DFK tri-peptide was inserted into the N-terminal region of g8p by two subsequent steps. First, fUSE5-ZZ DNA was amplified by PCR using primers P8-DFK/R-FOR (5'-CTGACTTTARGGGTCCTGCAGAAGCGGCCTTTGACTCCC-3') with M13g3*Bam*HI-REV (5'-TATTCACAAACGAATGGATCC 3') and in a second reaction using primers P8-DFK/R-REV (CAGGACCCRTAAAGTCAGCGAAAGACAGCATCGGAACG-3') with P5-*Bsr*GI-FOR (5'-TCGTCAGGGCAAGCCTTATTC-3'). Second, the resulting DNA fragments were assembled using primers P5-*Bsr*GI-FOR and M13g3*Bam*HI-REV. The assembled PCR product was purified, digested with restriction enzymes *Bsr*GI and *Bam*HI, and cloned into a similarly digested fUSE5-ZZ phage vector. The resulting phage was named fUSE5-ZZ-(p8)DFK. The g8p major coat protein of fUSE5-ZZ-(p8)DFK contains an amino terminal aspartate for drug conjugation by ECD chemistry to its carboxyl residue followed by the cathepsin-B cleavage site phenylalanine-Lysine (FK). In addition, it contains mutations that eliminated almost all of the naturally occurring free carboxyl groups on g8p that may be susceptible to EDC conjugation.

### Drug conjugation to phages by the EDC chemistry

The phage major coat protein g8p contains 3 carboxylic amino acid (glu2; Asp4; asp5;) that can be conjugated by application of (1-Ethyl-3- [3-dimethylaminopropyl] carbodiimide (EDC) chemistry, a rapid reaction performed at mild acidic pH (4.5–5.5) [60]. All the conjugations were done within a total volume of 1 ml of 0.1 M Na-citrate buffer; pH = 5, 0.75 M NaCl, 2.5 × 10^-6 ^mol of the aminoglycoside, 5 × 10^12 ^and phages that were already complexed with the targeting IgG. The reaction was initiated by the addition of 2.5 × 10^-6 ^mol of EDC, which was repeated two more times at time intervals of 30 min. Reactions were carried out at room temperature (~22°C) with gentle stirring in 2 ml Eppendorf tubes for a total of 2 h. The targeted drug-carrying phage nanoparticles were separated from the reactants by two successive dialysis steps of 16 h each against 1000 volumes of sterile 0.3 M NaCl.

### Quantifying linked Doxorubicin molecules/phage by cathepsin-B cleavage in a cell-free system

2 × 10^12 ^doxorubicin-conjugated fUSE5-ZZ-(g8p)DFK phages were suspended within 300 μl cathepsin-B reaction buffer as described [48]. Next, 7 units of cathepsin-B (Sigma, Israel) were added for 24 h at 37°. The phages were precipitated with PEG/NaCl and the supernatant was analyzed by reverse-phase HPLC and MALDI-TOF MS. For the HPLC analysis, a reverse phase C-18 column was used on a Waters machine with a gradient 0% to 100% of acetonitrile in the mobile phase, at 1 ml/min flow rate. Under these conditions, the doxorubicin containing peak at 480 nm was eluted 24–25 min after sample injection.

### Evaluation of target cell binding by whole-cell ELISA

Unless stated otherwise, all secondary antibodies, HRP-conjugated or fluorescent were from Jackson Immunoresearch Laboratories (USA). Evaluation of the binding ability of fUSE5-ZZ phages complexed with chFRP5 or trastuzumab to SKBR3 cells was done by whole-cell ELISA [36]. Following trypsinization, cells were washed once with 2% foetal calf serum, in PBS (incubation buffer, pH 7.4). In each experiment approximately 10^6 ^cells were divided into individual immunotubes (Nunc, Sweden). To confirm the specificity, phages (10^10 ^and 10^11 ^phage/ml) were added to the cell tubes for 1.5 h at 4°C. After washing ×2 with incubation buffer, HRP-conjugated rabbit anti M13 antibodies (GE healthcare, USA, 1/5000 dilution) were added to the immunotubes for 1 h at 4°C. Detection of cell bound phage was performed by addition of 0.5 ml of the chromogenic HRP substrate TMB (Dako, USA) to each tube and colour development was terminated with 0.25 ml of 1 M H_2_SO_4_. Finally, the tubes were centrifuged for 10 min at 4000 rpm and colour intensity of supernatants was measured at 450 nm.

### Analysis of phage internalization using confocal microscopy

Internalization of IgG-complexed phages into SKBR3 and A431 cells was studied using confocal microscopy as follows: Cells were grown on 24 mm cover slips in DMEM supplemented with 10% FCS essentially as described [36]. Subsequently, the medium was replaced by 450 μl DMEM without FCS into which 5 × 10^8 ^phages were added in 50 μl. After 3 h incubation at 37°C, the cells were gently washed ×3 with PBS and 100 μl of 1 μg/ml of membrane labelling CM-Dil (Molecular probes, USA) were added following incubation for 5 min at 37°C and 15 min at 4°C. Next, the cells were washed ×3 with PBS and fixed by 30 min incubation with 500 μl of 4% formaldehyde followed by washing with 1 ml PBS. To ensure efficient cell permeability, cells were washed with 250 μl of 0.2% triton × 100 in PBS, after which cells were blocked with 90% FCS in PBS containing 0.05% Tween 20 (Sigma, Israel) for 30 min. The blocking solution was aspirated and monoclonal mouse anti-M13 (1/100 dilution; GE healthcare, USA) was added for 1 h incubation following ×2 washes with 2% BSA in PBS. Subsequently, cells were incubated with 1:100 diluted FITC conjugated goat anti-mouse IgG for 1 h at room temperature. Finally, the cells were gently washed ×3 with PBS and images were acquired using a LSM 510 laser scanning confocal microscope (Vontz 3403B).

### Cell viability assay

The *in-vitro *cell-killing activity of hygromycin carrying fUSE5-ZZ-chFRP5 or doxorubicin carrying fUSE5-ZZ-(g8p)DFK-trastuzumab and fUSE5-ZZ-(g8p)DFK-cetuximab antibody complexed phage nanoparticles was measured by an MTT assay. Human breast carcinoma A431, SKBR3 target cells, or HEK293 control cells were seeded in 96-well plates at a density of 10^4 ^cells/well in DMEM supplemented with 10% FCS. |Targeted drug carrying phage nano-particles and relevant control phages were added to samples in 100 μl containing 5 × 10^11 ^phages and serial three-fold dilutions thereof and the cells were incubated at 37°C in 5% CO_2 _atmosphere. 48 h later, the media was replaced by phage-free media (100 μl per well) containing 5 mg/ml MTT reagent (Thiazolyl Blue Tetrazoliam Bromide, Sigma, Israel, dissolved in PBS) and the cells were incubated for another 4 h. MTT-formazan crystals were dissolved by the addition of 20% SDS, 50% DMF, pH 4.7 (100 μl per well) and incubation for 16 h at 37°C in 5% CO_2 _atmosphere. Absorbance at 570 nm was determined on a microtiter plate reader. Identical concentrations and combinations were tested in four separate wells per assay and the assay was performed at least three times. The results were expressed as percentage of living cells in comparison to the untreated controls that were processed simultaneously using the following equation: (*A*_570 _of treated sample/*A*_570 _of untreated sample) ×100.

## Authors' contributions

HB designed and carried out experiments and analyzed the data; IY designed and carried out experiments, participated in analyzing the data and in writing the manuscript. IB designed the study and wrote the manuscript.
